# Modulation of Apoptosis and Autophagy by Melatonin in Juglone-Exposed Bovine Oocytes

**DOI:** 10.3390/ani13091475

**Published:** 2023-04-26

**Authors:** Marwa El-Sheikh, Ahmed Atef Mesalam, Seon-Min Kang, Myeong-Don Joo, Seham Samir Soliman, Atif Ali Khan Khalil, Mi-Jeong Ahn, Il-Keun Kong

**Affiliations:** 1 Department of Microbial Biotechnology, Biotechnology Research Institute, National Research Centre (NRC), Dokki, Cairo 12622, Egypt; 2 Department of Therapeutic Chemistry, Pharmaceutical and Drug Industries Research Institute, National Research Centre (NRC), Dokki, Cairo 12622, Egypt; 3 Division of Applied Life Science (BK21 Four), Gyeongsang National University, Jinju 52828, Republic of Korea; 4 Department of Animal Reproduction and Artificial Insemination, Veterinary Research Institute, National Research Centre (NRC), Dokki, Cairo 12622, Egypt; 5 Department of Pharmacognosy, Faculty of Pharmaceutical and Allied Health Sciences, Lahore College for Women University, Lahore 54000, Pakistan; 6 College of Pharmacy and Research Institute of Pharmaceutical Sciences, Gyeongsang National University, Jinju 52828, Republic of Korea; 7 Institute of Agriculture and Life Science, Gyeongsang National University, Jinju 52828, Republic of Korea; 8 The King Kong Corp. Ltd., Gyeongsang National University, Jinju 52828, Republic of Korea

**Keywords:** melatonin, juglone, oxidative stress, developmental competence

## Abstract

**Simple Summary:**

During the in vitro embryo production (IVP) of embryos, the in vitro maturation (IVM) environment triggers the accumulation of reactive oxygen species that harm the maturation of oocytes and subsequent developmental competence. We used a bovine oocyte model to check the effect of melatonin (MT) supplementation during IVM on the quality and developmental competence of oocytes while under the stress of juglone. Using different analytical tools, the efficiency of MT in protecting oocytes from the deleterious effects of juglone-induced oxidative stress in bovine oocytes via the regulation of oocyte development, apoptosis, autophagy, and mitochondrial function was observed.

**Abstract:**

Melatonin, an antioxidant hormone secreted by the pineal gland, has been recognized as a regulator for numerous biological events. The deleterious effects of juglone, a polyphenolic extract of walnut trees, on embryo development has been previously reported. In the current study, we aimed to display the impact of melatonin administrated during in vitro oocyte maturation (IVM) on juglone-treated oocytes. Thus, in vitro matured oocytes were collected after 24 h post incubation with juglone in the presence or absence of melatonin. Reactive oxygen species (ROS), glutathione (GSH) content, mitochondrial distribution, and the relative abundance of mRNA transcription levels were assessed in oocytes, in addition, oocytes were in vitro fertilized to check the competency levels of oocytes to generate embryos. We found that administration of melatonin during the maturation of oocytes under juglone stress significantly improved the cleavage rate, 8-16 cell-stage embryos and day-8 blastocysts when compared to the sole juglone treatment. In addition, the fluorescence intensity of ROS increased, whereas the GSH decreased in juglone-treated oocytes compared to melatonin–juglone co-treated and untreated ones. Additionally, a significant increase in the mitochondrial aberrant pattern, the pattern that was normalized following melatonin supplementation, was observed following juglone administration. The mRNA analysis using RT-qPCR revealed a significant upregulation of autophagy and oxidative-stress-specific markers in the juglone-treated group compared to the co-treatment and control. In conclusion, the study reveals, for the first time, a protective effect of melatonin against the oxidative stress initiated following juglone treatment during the in vitro maturation of oocytes.

## 1. Introduction

Transferring the natural arrested oocyte from the stage of germinal vesicle (GV) to the metaphase II (MII) is the circumstance of oocyte maturation. The oocyte gains the capacity to produce an activation response, which is triggered by sperm during fertilization, and begins the future embryo development throughout this phase [1]. The ability of oocytes to develop embryos is mainly dependent on the quality of the oocyte and the maturation conditions, i.e., the environment in which the oocyte develops. The crucial processes that influence various key parameters include cumulus expansion, chromosomal condensation, progression to MII-stage, and the post-IVM developmental capacity of oocytes [1,2].

Apoptosis and autophagy are both the most common evolutionarily conserved machinery controlling cell fate. During these processes, different mechanisms become highly organized for the normal physiology and precise development of biological cells. In spite of the evident difference between the two, they are intimately connected and their regulatory events are share the same parameters [3].

Mitochondria are key organelles for sustaining the quality and functionality of oocytes since they serve as a primary source of ATP, and their dysfunctionality contributes to the aging of oocytes [4]. The mitochondrial pathway of apoptosis is controlled by the BCL2 protein family and the BCL2-associated X apoptosis regulator (BAX), which is the regulator of intrinsic apoptotic machinery [3]. BAX is mostly present in the mitochondrial cytosol as an inactive monomer, and during the apoptotic pathway, it mediates the release of cytochrome c, which is a mitochondria-mediated apoptosis marker, from the intermembrane space into the cytosol [5].

Additionally, mitochondrial dynamics are controlled by the fission and fusion of the mitochondria, the two processes that are responsible for the generation of new mitochondria and the elimination of old and damaged ones [6]. The reduction in mitochondrial fission commonly contributes to an inhibition of apoptosis and promotion of cell survival [7]. The dynamin-related protein 1 (Drp1) is the pro-fission marker, and the regulation of mitochondrial fission is adjusted by an apoptotic BAX marker, while Drp1 may increase BAX oligomerization, the essential process for permeabilization of mitochondrial outer membranes [8,9].

The in vitro maturation (IVM) environments usually trigger an increase in the oxygen content, causing the formation of significant amounts of reactive oxygen species (ROS), which can harm matured oocytes and, subsequently, the growing embryos. ROS is one of the main reasons for faulty gametes or underdeveloped embryos in assisted reproductive technology (ART) and are shielded from by the administration of different antioxidants such as melatonin (MT) [10,11,12].

MT is a natural hormone secreted by the pineal gland of vertebrates. Besides having anti-apoptotic properties, MT is considered an immunomodulatory and cytoprotective agent, and it was confirmed to be synthesized by the cumulus–oocyte complexes (COC) in bovines [13]. In ART, melatonin’s antioxidant activity has been shown to improve the nuclear maturation and quality of inferior and non-inferior oocytes in addition to its ability to protect against several harmful substances [10,11,12,13,14]; moreover, it also reduced oxidative stress in bovine ovarian granulosa cells [12]. In this concern, a similar pattern of results was previously reported to indicate the efficacy of melatonin when treated in vitro during oocyte maturation in the enhancement of the quality of oocytes and development of embryos [10,11,13,15]. Furthermore, MT supplementation during IVM under the stress of different toxic compounds significantly improved oocyte maturation and downregulated the levels of oxidative stress in oocytes and embryos [10,11].

Juglone, also called 5-hydroxy-1,4-naphthalenedione, is the polyphenolic extract of walnut trees (Juglans). It is a naturally occurring compound that has a various biological functions including the immunoregulatory, anti-inflammatory, antimicrobial, and anticancer properties [16]. Juglone’s impact on cancer cells was manifested through several mechanisms such as blocking pathways for tumor cell proliferation and migration, in addition to the induction of apoptosis and autophagy. Juglone exerts its effects on the biological cell via the interaction with various signaling pathways, such as the PI3K/AKT/mTOR, mitogen-activated protein kinases (MAPK), IL-6/STAT3, and AMP-activated protein kinase (AMPK) [16,17,18]. During oocyte maturation, the deleterious effects of juglone were reported as the decline in the percentage of oocytes at metaphase II stage as well as the induction of both apoptosis and autophagy in matured oocytes [19]. Additionally, juglone-treated oocytes displayed a downregulation in the markers of the PI3K/AKT/mTOR signaling pathway [18]. Thus, in the current study we aimed to display the effect of melatonin administration during oocyte maturation under the stress of juglone. The concentration of juglone was selected based on our earlier data that explored that juglone at a 12.5–50 µM dose significantly induced oxidative stress in oocytes and retarded the cleavage and blastocyst development rates [19]. To our knowledge, this is the first study to reveal the interplay of a juglone–MT co-treatment during in vitro maturation (IVM) and the post-fertilization of bovine oocytes. To study our hypothesis, the effects of MT, treated during IVM, on bovine oocytes and under the stress of juglone were checked. Different parameters regulating oocyte development, oxidative stress, and mitochondrial function were inspected, and in addition, the developmental potential of oocytes to generate embryos has been checked upon exposure to juglone and juglone–MT co-treatment.

## 2. Materials and Methods

### 2.1. Experimental Setup

The current study was performed to check the effects of melatonin application in the presence of juglone treatment on the quality of oocytes and their developmental potential. In our previous investigations, we elucidated the toxicity of juglone on bovine oocytes when administrated during IVM [19]. We also explored the efficiency of melatonin to retract the damaging effects of the anti-developmental SH6 compound by improving the different parameters regulating oocyte growth and embryo development [10]. The first experiment in the present study was performed to check the effects of melatonin treated during IVM for 24 h in the presence of juglone 20 µM. Serial dilutions of melatonin were tested (10^−9^, 10^−8^, 10^−7^ M) and were compared to the sole juglone-treated, and untreated control groups. Using a quantity of 8–16 cells, cleavage and blastocyst development rates were counted under the stereomicroscope (Olympus SZ51, Tokyo, Japan). The second experiment was carried out on three groups, including the juglone 20 μM, the co-treated melatonin 10^−7^ M- juglone 20 μM combination groups, and the control group. The effects of melatonin administration on the different parameters including oxidative stress, glutathione content, autophagy, and mitochondrial function were examined.

### 2.2. Reagents and Eithical Statement

The Study experimental techniques and procedures were approved based on the Institutional Animal care and Use Committee (GAR-110502-X0017). Reagents and chemicals used in the current study were obtained from Sigma-Aldrich (St. Louis, MO, USA) unless otherwise mentioned.

### 2.3. Oocytes Aspiration and Collection

Bovine ovaries were collected from an abattoir and then transported within 2 h after slaughter to the laboratory. Oocytes were washed in Dulbecco’s phosphate-buffered saline (D-PBS) that was fresh and pre-warmed before the experiment. Cumulus–oocyte complexes (COCs) were aspirated and picked up under stereomicroscope in TL-HEPES medium (10 mM HEPES, 0.34 mM sodium biphosphate, 114 mM sodium chloride, 2 mM sodium bicarbonate, 10 mM sodium lactate, 3.2 mM potassium chloride, 2.0 mM calcium chloride, 0.5 mM magnesium chloride, 1 μL/mL phenol red, 0.1 mg/mL streptomycin and 100 IU/mL penicillin).

### 2.4. In Vitro Maturation (IVM) and Biochemical Treatment

Collected COCs were then washed several times using IVM medium (TCM-199 supplemented with 10 µg/mL follicle-stimulating hormone (FSH), 10% fetal bovine serum (FBS; Gibco BRL, Life Technologies, Grand Island, NY, USA), 1 µg/mL estradiol-17ß, 10 ng/mL epidermal growth factor (EGF), and 0.2 mM sodium pyruvate and 0.6 mM cysteine). Groups of around 50 COCs were cultured for 24 h in 500 μL of IVM medium in four-well plates (Thermo Fisher Scientific, Waltham, MA, USA) in the presence or absence of 20 µM of juglone and/or melatonin and incubated at 38.5 °C under 5% CO_2_.

### 2.5. In Vitro Fertilization and In Vitro Embryo Development

After 24 h post maturation, oocytes were used for in vitro fertilization (IVF) using frozen bovine semen. In brief, cryopreserved sperm straws were directly thawed for 1 min at 38 °C and diluted in pre-warmed DPBS, then centrifuged at 750× *g* for 5 min at room temperature. Following centrifugation, the sperm pellets were resuspended in heparin (20 μg/mL) prepared in IVF medium (Sodium pyruvate (22 mg/mL), Tyrode’s lactate solution, bovine serum albumin (BSA; 6 mg/mL), streptomycin (0.1 mg/mL), and penicillin (100 IU/mL)) and incubated at 38.5 °C for 15 min. The concerted sperm were then diluted with IVF medium to a final density of 1 × 10^6^ sperm/mL that was used for oocytes enrichment by adding 700 μL to each oocytes group; the two mixtures were incubated for 18–20 h at 38.5 °C and under humidified conditions of 5% CO_2_.

The day following IVF (Day = 1), the cumulus cells were removed from oocytes by successive pipetting, following by oocyte washing in SOF-BE1-SOF + BSA + ITS medium (BSA (4 mg/mL), insulin (5 μg/mL), transferrin (5 μg/mL)m and sodium selenite (5 ng/mL). Then, presumptive zygotes were incubated by 700 μL SOF + BSA + ITS medium in a 4-well plate and kept at 38.5 °C under 5% CO_2_. On day 4 following IVF, we recorded the total number of cleaved embryos, with the 8-cell stage embryos to be further cultured in renewed medium 4 days later. On day 8 post fertilization, blastocysts were collected after being washed several times in TL-HEPES. The embryos were either stored at −80 °C after being frozen using liquid nitrogen or kept at 4 °C following washing several times in 4% paraformaldehyde (PFA) prepared in PBS.

### 2.6. Quantification of Reactive Oxygen Species (ROS) and Glutathione (GSH) Content

Matured oocytes were collected, washed in PBS, and exposed to 5 μM of 2,7-dichlorodihydrofluorescein diacetate (H2DCFDA; the ROS indicator) for 20 min at 38.5 °C in humidified conditions (5% CO_2_). Treated samples were washed 3 times in PBS-PVA and spotted on glass slide to examine under a epifluorescence microscope at 525 nm emission and 490 nm excitation wavelengths. To estimate the GSH content, oocytes were incubated with 30 μM ThiolTracker Violet GSH stain (Thermo Fisher Scientific, Waltham, MA, USA) for 30 min at 38.5 °C then washed in PBS and visualized under epifluorescence microscope. The fluorescence intensities of ROS and GSH were estimated using ImageJ software (National Institutes of Health, Bethesda, MD, USA; https://imagej.nih.gov/ij/, accessed on 1 January 2023).

### 2.7. Evaluation of Mitochondrial Distribution

To investigate the mitochondrial distribution patterns in matured oocytes, the MitoTracker deep Red stain (Molecular Probes, Eugene, OR, USA) was used [20]. Briefly, 100 nM MitoTracker was incubated with matured oocytes for 40 min at 38.5 °C. Then, oocytes were washed in PBS-PVA and fixed using 4% paraformaldehyde (PFA) for 15 min. On glass slides, samples were spotted for examination under an epifluorescence microscope. The homogeneous distribution patterns were detected when the mitochondria were distributed throughout the whole oocyte cytoplasm, while aberrant patterns were displayed when mitochondria were dispersed either peripheral or semi peripheral in the cytoplasm.

### 2.8. Total RNA Extraction and cDNA Synthesis

Around 50 COCs per group were collected for total RNA extraction using the Arcturus PicoPure RNA Isolation Kit (Arcturus, Foster, CA, USA). The cDNA synthesis was performed by adding 5× iScript reaction mixture and iScript reverse transcriptase (4:1) and further mixed with the total volume of RNA. The cDNA was synthesized according to the following conditions: 25 °C, 5 min incubation time; 42 °C, 30 min for annealing; and 85 °C, 5 min for enzyme inactivation. The concentration of cDNA was detected using NanoDrop 2000c spectrophotometer (Thermo Fisher Scientific, Waltham, MA, USA) and stored at −20 °C until use for RT-qPCR.

### 2.9. Quantitative Reverse Transcription PCR (RT-qPCR)

While using iQ-SYBR GREEN Supermix according to manufacturer instructions, the stored cDNA was subjected for RT-qPCR analysis. In brief, cDNA and primer mixture were mixed with the iQ-SYBR GREEN Supermix. The selected primers in the study were either selected based on previous investigations or designed by the online primer3 software and according to the mRNA sequence of the definite bovine gene in the GenBank (these genes, their primers sequences, amplicons sizes are summarized in Table 1).

The condition of the qPCR reaction was started by heating at 95 °C for 3 min, then the initial denaturation step followed by 15 s at the same temperature for 44 cycles, then heating decreases until 58 °C for 20 s, followed by 72 °C for 30 s; the final extension was performed at 72 °C for 5 min. The reaction was proceeded using the CFX98 instrument (Bio-Rad Laboratories, Hercules, CA, USA).

The ΔΔCT method was used to check the abundance of each tested gene. The full names of all genes used in the current study are as follows: autophagy-related gene 5 and 7 (ATG5; ATG7), BCL2 interacting protein 3 (BNIP3), microtubule associated protein 1 light chain 3 alpha (MAP1LC3A, LC3A), microtubule associated protein 1 light chain 3 beta (MAP1LC3B, LC3B), autophagy-related gene 6 (Beclin-1), protein kinase B (PKB, AKT 2), B-cell lymphoma 2 (BCL2), superoxidase dismutase 1 (SOD1), dynamin-related protein 1 (Drp1), nuclear factor kappa B (NF-KB), BCL2 associated X apoptosis regulator (BAX), and the housekeeping gene (glyceraldehyde-3-phosphate dehydrogenase; GAPDH).

### 2.10. Statistical Analysis

Differences in developmental competence and the data of fluorescence intensity were analyzed using one-way ANOVA and multiple comparisons test. GraphPad Prism software version 6 was used to analyze the data. The mRNA transcription levels of selected genes were analyzed using an unpaired *t*-test. The images presented in the current study were explored using ImageJ software. Significance degree was presented as asterisk(s); *, **, ***, and **** when the *p* values were <0.05, 0.01, 0.001, and 0.0001, respectively. The data were tested for normal distribution and underwent a logarithmic transformation when this criterion was not met.

## 3. Results

### 3.1. Melatonin Application during IVM Improves the Developmental Competence of Juglone-Treated Oocytes

**Figure 1:**
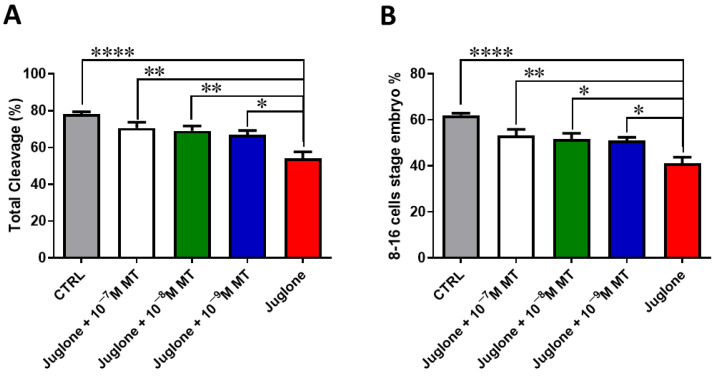
Effect of melatonin administration during IVM on the number of cleaved embryos of juglone-treated oocytes. Different concentrations of melatonin (10^−7^, 10^−8^, 10^−9^ M) were added during IVM in combination with 20 µM of juglone in addition to the sole juglone and untreated control groups. (**A**) Total cleavage rates and (**B**) the number of 8–16 cell stage embryos generated after treatment of oocytes with melatonin and/or juglone. Significance degree was presented as asterisk(s); *, **, and **** when the *p* values were <0.05, 0.01, and 0.0001, respectively.

**Figure 2:**
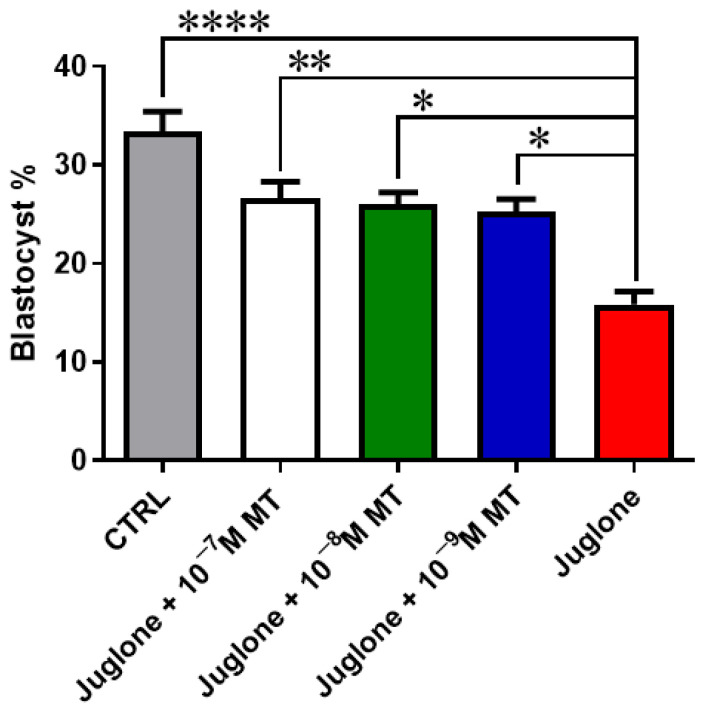
Embryo development post MT/juglone treatment. Different concentrations of melatonin (10^−7^, 10^−8^, 10^−9^ M) were incubated with oocytes at IVM under exposure to 20 µM of juglone in addition to the sole juglone and untreated control groups. Significance degree was presented as asterisk(s); *, **, and **** when the *p* values were <0.05, 0.01, and 0.0001, respectively.

Oocyte exposure to melatonin at 10^−7^, 10^−8^, and 10^−9^ M during oocyte maturation and in combination with 20 µM of juglone significantly improved the total number of cleaved and 8–16 cells stage embryos as seen in Figure 1. Moreover, co-incubation of juglone with melatonin at all tested doses significantly improved the percentage of developed embryos that recorded significant improvement in all experimental groups (Figure 2).

### 3.2. Juglone Treatment Negatively Affects, while Melatonin Abrogates, Oxidative Stress in Matured Oocytes

**Figure 3:**
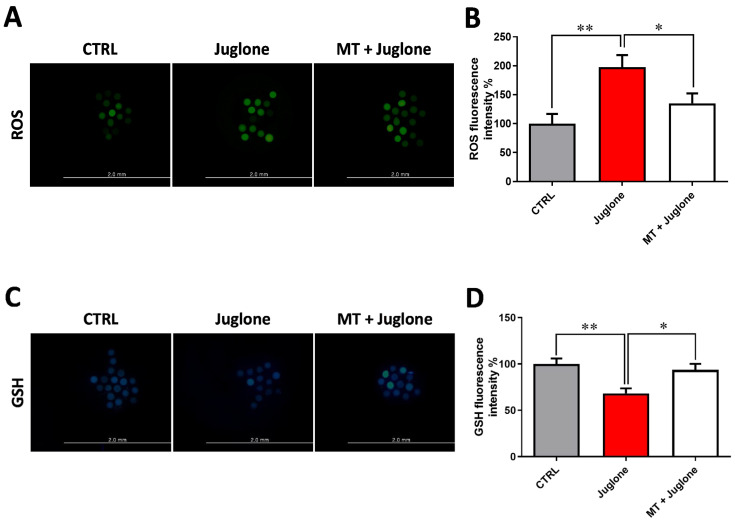
Impact of melatonin treatment during oocyte maturation on oxidative stress in juglone-exposed oocytes. Melatonin (10^−7^ M) was used to treat oocytes in the presence and absence of 20 µM of juglone and (**A**) matured oocytes were incubated with H_2_DCFDA to check the intracellular reactive oxygen species (ROS) in experimental groups; (**B**) ROS fluorescent intensity in control and treated groups analyzed by ImageJ software; (**C**) collected fluorescence images of glutathione (GSH) staining in matured oocytes; (**D**) quantification levels of GSH fluorescence intensity from different groups. Scale bar = 0.2 mm. Significance degree was presented as asterisk(s); *, and ** when the *p* values were <0.05, 0.01, respectively.

Due to the observed protective effects of melatonin being achieved in the combination group of melatonin 10^−7^ M and juglone 20 µM, all the subsequent experiments, in addition to the sole juglone 20 µM and control groups, were conducted using this combination group. To scrutinize the oxidative stress in oocytes, the GSH content and ROS levels were determined using ThiolTracker Violet and H_2_DCFDA staining, respectively. Fluorescence intensity of ROS and GSH was correspondingly increased and decreased in juglone-treated oocytes compared to melatonin-juglone co-treated and untreated groups that showed the opposite profiles (Figure 3A–D).

### 3.3. Juglone Treatment Induces, While Melatonin Restores, Mitochondrial Dysfunction, Apoptosis, and Autophagy in Matured Oocytes

**Figure 4:**
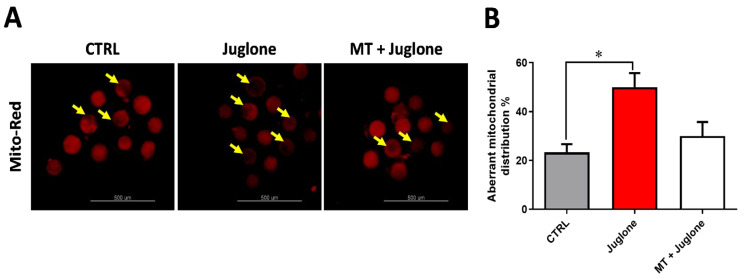
Assessment of melatonin and juglone effects on mitochondrial health in matured bovine oocytes. (**A**) Representative images of MitoTracker Red staining from different groups; (**B**) proportion of matured oocytes representing the aberrant distribution of mitochondria in each oocyte treatment group. Scale bar = 500 µm, and values with asterisk (*) indicating statistical significance (*p* < 0.05).

Furthermore, the impact of juglone and melatonin administration during IVM on mitochondrial distribution pattern was inspected using the MitoTracker Deep Red. We found a significant increase in the mitochondrial aberrant pattern in juglone treatment, while a lower incidence was detected in the other experimental groups (Figure 4A,B).

**Figure 5:**
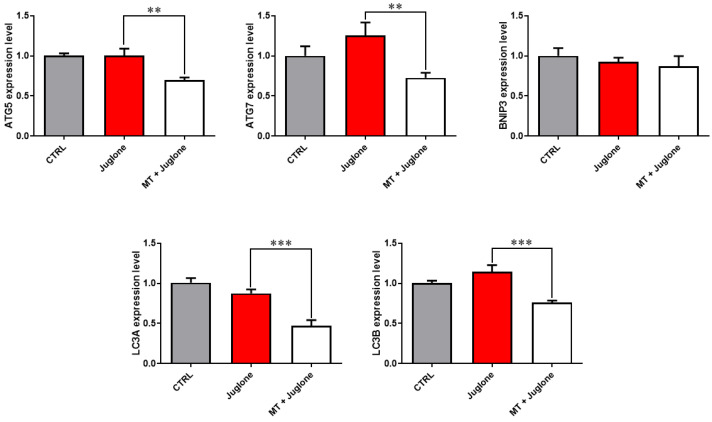
Relative expression of autophagy-specific genes in COCs. Asterisk indicates statistical significance. Significance degree was presented as asterisk(s); **, and *** when the *p* values were <0.01, and 0.001, respectively (Student’s *t*-test).

**Figure 6:**
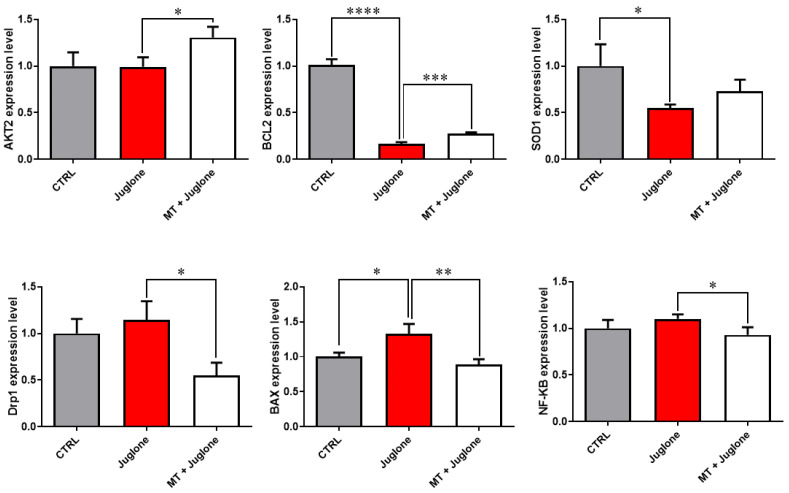
Relative expression of oxidative stress-related genes in COCs. Significance degree was presented as asterisk(s); *, **, ***, and **** when the *p* values were <0.05, 0.01, 0.001, and 0.0001, respectively (Student’s *t*-test).

Moreover, checking the mRNA expression levels of various markers involved in the regulation of autophagy in matured oocytes revealed a significant upregulation of the mRNA levels of the ATG5, ATG7, LC3A, and LC3B markers in the juglone-treated compared to the co-treatment group (Figure 5). In addition, as seen in Figure 6, the molecular characterization of oxidative-stress-related genes displayed a sharp decrease in mRNA levels of the anti-apoptotic gene BCL2, and melatonin significantly restored the expression levels. Low expression patterns in the levels of the mitochondrial fission Drp1, and oxidative-stress-related genes BAX and NF-KB were observed in melatonin–juglone dual treatment group compared to the sole juglone-exposed oocytes, while the expression of the mitochondrial AKT2 gene was upregulated in the co-treated melatonin and juglone oocytes.

## 4. Discussion

Melatonin (MT), a potent antioxidant, plays a critical role in the field of reproduction since it exists in high levels in follicular fluid, while the MT receptors exist on the surface of oocyte and cumulus cell [21]. Using a bovine cumulus–oocyte complex (COC) model, it was conveyed that the biosynthesis of melatonin in COCs in vitro play a critical role during the oocyte maturation process [13]. Nevertheless, the definite mechanisms underlying the functionality of melatonin are not fully identified. MT was previously shown to protect oocyte and embryos from the risk of nuclear fragmentation and oxidative stress in addition to its efficiency to improve oocyte maturation [13,22,23]. In our present study, the bovine oocyte model was used to check the impact of the antioxidant melatonin and a naturally produced juglone compound administrated during IVM on the quality and developmental competence of oocytes.

Juglone is the polyphenolic extract of walnut trees (Juglans), and it was considered an anti-inflammatory, immunoregulatory, antimicrobial, and anticancer agent [16]. In the context of embryo development, the effects of juglone during embryo development has been previously studied by us in an earlier investigation [19], however in ART, the potential interaction between MT and juglone has not yet been investigated. We observed that exposure of oocytes to MT at different concentrations ranging from 10^−7^, 10^−8^ to 10^−9^ M while under the stress of 20 µM juglone exposure significantly restored the developmental competence which was restricted in the sole juglone treatment. While the 10^−7^ M dose was the most effective in the improvement of all developmental processes. In line with our previous results, MT at the same concentration was reported to alleviate the toxicity of the dietary supplement nicotinamide when treated at high concentration during oocyte maturation [11]. Moreover, under the stress of mycotoxins (β-zearalenol and HT-2), MT has been reported to alleviated the toxicity of these compounds in bovine ovarian granulosa cells [12]. Additionally, the deleterious effects of bisphenol A, aflatoxin B1, and paraquat herbicide have been attenuated upon the treatment of MT during oocyte maturation [24,25,26].

Oocytes are widely perceived as the largest cell in mammals and different multicellular organisms [4]. The competency of oocytes to develop embryo is largely dependent on different internal and external variables, and the accumulation of ROS is one of the critical obstacles affects this process [1]. Checking the ROS levels in oocytes demonstrated the induction of the levels in juglone oocytes and MT has been successfully diminished ROS to levels that was closely similar to the control. Consistent with our results, in bovine oocytes, juglone was previously stated to induce ROS, whereas MT downregulated ROS, under the stress of anti-developmental compounds [10,11,19].

Glutathione (GSH) is a mechanism that exists in biological cells as an antioxidant defense against ROS. The synthesis of GSH during IVM has been showed to have a critical role in oocyte function and embryo development [27]. Herby, in the current study we detected an obvious increase in the GSH in oocytes co-treated with MT and juglone, unlike the clear reduction observed in the sole juglone treatment, supporting our abovementioned fundings regarding the decrease in ROS and the enhancement of developmental competence in the co-treatment MT and juglone setups.

Mitochondria are key organelles for the different biological processes such as energy production, and cellular adaptation to stressors including oxidative stress and DNA damage. In the current study, we checked the mitochondrial distribution pattern in matured oocytes. We noticed a dramatic induction in the aberrant distribution of mitochondria in juglone-treated oocytes, while the opposite profile was detected in the MT–Juglone co-treated and control oocytes. In line with our results, MT efficacy to restore the normal distribution pattern of mitochondria in oocytes was previously reported [10]. In another study, melatonin successfully maintained the homogenous mitochondrial distribution in oocytes that were treated with a high concentration of Nicotinamide during IVM [11]. Additionally, MT was recently reported to protect human oocytes in prolonged cryopreservation by improving mitochondrial function and maintaining the normal developmental competence and ROS/GSH balance [28], which is in line with our abovementioned results.

The ability of mitochondria to change their shape is a process referred to as mitochondrial dynamics, which is regulated by the fusion and fission of the outer/inner mitochondrial membranes [6]. The fission of mitochondria is commonly related to mitochondrial dysfunction, and the decrease in mitochondrial fission inhibits apoptosis and promotes cell survival [7]. The dynamin-related protein 1 (Drp1), a well-known pro-fission marker, is responsible for the clearance of damaged mitochondria via mitophagy, i.e., mitochondria-selective autophagy [6]. Our results demonstrated the upregulation in the mRNA expression level of Drp1 in juglone-exposed oocytes, while melatonin significantly decreased Drp1 expression level, suggesting the role of MT in the modulation of mitophagy under unfavorable conditions. Recently, Qu et al. has been reported that MT protects mouse oocytes from the cadmium-based environmental pollutant [29], an effect that the authors demonstrates by the ability of MT to change epigenetic modification, besides the enhancement of the function and morphology of mitochondrion. This finding affirms the cytoprotective ability of MT and supports our data on the effect of MT on oocyte mitochondria.

Autophagy is the process by which the cell can control apoptosis and removes the damaged substances and abnormal proteins in its cytoplasm. During IVM, the hyperactivation of autophagy was reported to have a negative impact on oocyte quality, the similar effects that are caused by the induction of apoptosis in oocyte [11,20]. We and others previously displayed the induction of both apoptosis and autophagy by juglone via the interaction with the different cellular signaling pathways such as the PI3K/AKT/mTOR in bovine oocytes and the MAPK pathway in hepatocellular carcinoma [18,30]. Interestingly, the administration of MT during IVM and in the presence of different toxic compounds was confirmed to have a great impact on the downregulation of the levels of both apoptosis and autophagy in bovine oocytes [10,11]. In the current study, we demonstrated the efficiency of MT to diminish the expression of various genes related to autophagy under the stress of juglone-induced autophagy in oocytes, and these results confirm the previously mentioned investigations in this concern. Moreover, it affirms the earlier studies on the interfering impact of both apoptosis and autophagy on the developmental competence of bovine oocyte and mitochondrial health [20,31]. These data also confirm our abovementioned finding of the decrease in developmental competence and homogenous distribution pattern of mitochondria during juglone treatment, effects which were suppressed in the MT-Juglone co-treatment experiment

It is noteworthy to mention that the anti-apoptotic BCL2 gene family, such as the BCL2 and BAX, was reported to block autophagy by binding with the autophagy-related protein 5 (ATG5), which highlights the crosstalk between autophagy and apoptosis. ATG5 have the ability to regulate the components of the extrinsic apoptosis and the knockdown of ATG5 was reported to protect cancer cells from apoptosis [3]. Our results explored the induction in the expression pattern of ATG5, ATG7, LC3A, LC3B autophagy and BAX apoptotic markers in the juglone treatment, and MT significantly modulated these levels. Moreover, juglone-exposed oocytes have showed a significant downregulation in BCL2 transcription, while the opposite profile was detected upon the addition of MT, endorsing the ability of MT to eliminate the toxicity of juglone on oocytes through apoptosis- and autophagy-attenuating potential. This also agrees with our results of the unfavorable induction in ROS and the clear decrease in the GSH content in juglone-exposed oocytes, while the reverse effect was noticed following the application of MT.

## 5. Conclusions

Collectively, the current study shows the potential deleterious effects of juglone at high concentrations on preimplantation embryo development. Furthermore, to the best of our knowledge, we are the first to indicate the efficacy of melatonin to shield oocytes and presumptive zygotes from the deleterious effects of juglone treated during oocyte maturation. The effects that were achieved via the antioxidant activity of melatonin contracted the levels of both apoptosis and autophagy in juglone-exposed oocytes. Likewise, the restorative and protective effects of melatonin makes it a potentially convenient approach in ART.

## Figures and Tables

**Table 1:** The names of the tested genes, sequences of primers, sizes of PCR products, and accession numbers for RT-qPCR experiments.

Gene Name	Sequence	Amplicon Size (bp)	Accession Number
ATG5	F: CCACTGCCGTCATTAAACCT R: TTCCACTCCCTCGAGCTAAA	212	XM_027551305.1
ATG7	F: ATGGCCTTTGAGGAACCTTT R: ATGCCTCCCTTCTGGTTCTT	210	XM_010817935.3
BNIP3	F: GAAGGAATGCCGACACTAGG R: CAAAGCCAGCAGACACTCAG	138	XM_027528566.1
LC3A	F: CATGAGCGAGTTGGTCAAAA R: GGGAGGCGTAGACCATGTAG	170	XM_027558753.1
LC3B	F: TTATCCGAGAGCAGCATCC R: AGGCTTGATTAGCATTGAGC	171	XM_027513856.1
AKT2	F: CGACTATCTCAAACTCCTGG R: ATCTTCATGGCATAGTAGCG	90	NM_001206146.2
BCL2	F: TGGATGACCGAGTACCTGAA R: CAGCCAGGAGAAATCAAACA	120	NM_001166486.1
SOD1	F: CCATCCACTTCGAGGCAAAG R: TCTCCAAACTGATGGACGTGG	100	NM_174615.2
Drp1	F: AGCCCCTTCTAGAGCAGTGG R: CGGGTGACAATACCAGTGCC	89	XM_024992090.1
BAX	F: CACCAAGAAGCTGAGCGAGTGT R: TCGGAAAAAGACCTCTCGGGGA	118	NM_173894
NF-KB	F: TGGCGGAATTACCTTCCATAC R: CATCACTCTTGCCACAACTTTC	110	DQ464067
GADPH	F: CCCAGAATATCATCCCTGCT R: CTGCTTCACCACCTTCTTGA	185	NM_001034034.2

F, forward; R, reverse.

## Data Availability

All of the data is contained within the article.
